# What does the brain tell us about abstract art?

**DOI:** 10.3389/fnhum.2014.00085

**Published:** 2014-02-28

**Authors:** Vered Aviv

**Affiliations:** Faculty of Dance, The Jerusalem Academy of Music and Dance,Jerusalem, Israel

**Keywords:** abstract art, neuroesthetics, neural correlates of art, artistic preference, art and associations

## Abstract

In this essay I focus on the question of why we are attracted to abstract art (perhaps more accurately, non-representational or object-free art). After elaborating on the processing of visual art in general and abstract art in particular, I discuss recent data from neuroscience and behavioral studies related to abstract art. I conclude with several speculations concerning our apparent appeal to this particular type of art. In particular, I claim that abstract art frees our brain from the dominance of reality, enabling it to flow within its inner states, create new emotional and cognitive associations, and activate brain-states that are otherwise harder to access. This process is apparently rewarding as it enables the exploration of yet undiscovered inner territories of the viewer’s brain.

## Art and reality

Over the course of human evolution, the phenomenon of art appeared some 30,000 years ago and humans became increasingly occupied with creating and appreciating works of art (Humphery, 1999; Solso, 1999). Art works are sensed and perceived via the same neuronal machinery and anatomical routes that were primarily developed for interacting with, and comprehending, “reality”. These mechanisms evolved in order for us to acquire and analyze sensory information from the world around us and, consequently, to successfully and adaptively behave in an ever-changing environment (see the “Perception Action loop” theory in Tishby and Polani, 2011).

The visual system, which is the vehicle that processes visual art, is aimed at filtering, organizing and putting (functional) order to the enormous amount of data streaming into our visual system. Interestingly, at early stages of visual processing, the visual scene is deconstructed into its elementary components such as spots of light, lines, edges, simple forms, colors, movement, etc. At later (higher) stages, the system reconstructs these components into complicated forms and objects: a moving car, a face with blinking eyes, a pirouette of a dancer (Zeki, 1992; Hubel, 1998). Being an efficient learning machine, our brain uses bidirectional (“top down” and “bottom up”) processing schemes and algorithms for visual scene analysis. Namely, we first build (predict) a tentative model, an optional representation, of the visual world and this model is then verified and updated with increased accuracy against the “evidence” presented by the sensory stimulus (Hochstein and Ahissar, 2002; Bar, 2007; Tishby and Polani, 2011). These ongoing bidirectional processes enable us to make quick and effective generalizations and decisions about the world.

In contrast to the processing of daily objects, art is free from the functional restrictions imposed on the visual system during our daily life. Art is very often engaged in finding new ways to organize and represent objects and scenery. Artists are liberated to represent and to decompose depicted objects in various non-functional (non –“realistic”) ways. Examples are works by artists of the Cubist (e.g., George Braque and Pablo Picasso) or Surrealist (e.g., Salvador Dali and Juan Miro) movements. Artworks could also be only partially faithful representations of our daily visual experience, such as the monochromatic blue figures of Pablo Picasso or the blue horses of Franz Marc, and it can be “free” from obeying the laws of physics (e.g., the flying figures of Marc Chagall or the impossible objects of E.C. Escher). Apparently we categorize some inputs as artworks while others as non-art. We make this distinction based on contextual, cultural and perceptual parameters. Interestingly, a major distinction between perceiving an object as piece of art or as part of the daily visual (non-art) experience, relies on the presence of artistic style (such as the brush work of the painter) and not only on the content of the scene (Augustin et al., 2008; Cupchik et al., 2009; and see also Cavanagh and Perdreau, 2011; Di Dio et al., 2011).

The above notion brings to mind the unique character of abstract art, which, unlike representational art and other forms of art mentioned above, does not exemplify objects or entities familiar to our visual system during daily life experience. Still, as all visual information, abstract art is perceived via the same system that was developed primarily in order to functionally represent real-world objects. This places abstract art in a unique position within visual processing—far from the natural (“survival”) role of that system. It is therefore intriguing to try and understand why we are attracted to abstract art (as demonstrated by the huge success of museum exhibitions of the abstract artwork, such as those of Jackson Pollock). This must mean that abstract art, which is a rather new human invention, offers something attractive to the viewer’s brain. So I would like to ask: what does abstract art offer to the viewer’s mind?

It should be noted that this article focuses on the two ends of a continuum between representational art and abstract art, and therefore not relating to the in-between category of paintings, i.e., semi-representational or semi-abstract works.

## Neural and behavioral correlates of art/abstract art

A fundamental assumption of modern brain research is that each action in mental/cognitive/emotional realms is correlated with a corresponding specific brain activity pattern. Each activity represents and generates the resultant experience. It is therefore worth seeking for the neural correlates of the abstract art experience and attempting to extract the principles underlying the neural processing of this form of art.

In an fMRI imaging study, Kawabata and Zeki (2004) demonstrated that different categories of painting—landscape, portrait and still life—evoked activity at localized and category-specific brain regions. In contrast, abstract art did not activate a unique localized brain region. Rather, brain activity related to abstract art appeared in brain regions activated by all other categories as well. Thus, when subtracting the fMRI signal generated by abstract art from signals generated by representative art of the various types (landscape, portraits, still life) then zero activity was observed.

This is surprising as one might assume that there would be neural correlates (i.e., specific brain activity) for the specific cognitive category recognition of abstract art. On the other hand, because abstract art does not consist of clear well-characterized objects, but rather is composed of basic visual elements such as lines, spots, color patches and simple forms such as triangles, one might expect the activity corresponding to these basic elements to also appear in other categories of brain activity. In this case, we should not expect a unique brain activity related to abstract art as indeed was found by Kawabata and Zeki (2004) as well as by Vartanian and Goel (2004). To put it differently, it seems that we know that we view abstract art by realizing that what we view does not belong to any other specific category of art. Namely, we recognize abstract art by exclusion.

In addition to fMRI studies, abstract art was also studied by behavioral and by direct voltage electroencephalogaphy (DC-EEG) methods. Combining behavioral and low-resolution electromagnetic tomography analysis, Lengger et al. (2007) demonstrated that observers preferred abstract and representational paintings in an equal manner. Yet the abstract stimuli evoked more positive emotions. Representational artworks were classified as more interesting, were understood better and induced more associations (as reported subjectively by the observers). Information about the painting (such as the title of the paining, the artist’s name, the technique used) increased understanding of each style (representational as well as abstract art), but it did not change other parameters of evaluations (i.e., preference, associations, emotions). Comparing brain activity in response to representational and abstract paintings revealed significantly higher activation for representational art works in several brain regions, predominantly in the left frontal lobe and bilaterally in the temporal, frontal and parietal lobes, limbic system, insula and other areas as well. Increased brain activity in response to representational art was mostly attributed to the process of object recognition, and the activation of memory and associations systems. Introducing stylistic information seemed to reduce cortical activation, for both representational and abstract art. The authors concluded that information on artworks seems to facilitate the neural processing of the stimuli.

The idea that knowledge and experience facilitate the processing of the visual stimuli was also evident in the work of Solso (2000). Solso monitored brain activity of a portrait-artist (via fMRI) while he drew faces, and compared the artist’s brain activity with that of a non-artist who was drawing the same faces. Brain activity of the artist revealed less activity in face processing areas (posterior parietal) than that of the non-artists. This lower level of activation of the artist’s face recognition area indicates that he may be more efficient in the processing of facial features than the novice.

From the above experiments one may conclude that abstract art, stylistic knowledge and experience all seems to reduce cortical brain activity as compared to the relevant controls (representational art, stylistic knowledge and novice, correspondingly). These results indicate that the analysis of abstract art evokes less focal brain activation.

The study by Vartanian and Goel (2004), presents some evidence that a reduction in subjective aesthetic preference is correlated with decreased activity in certain brain areas involved with reward systems, whereas greater aesthetic preference evoked larger activity in other brain areas, involved with emotional valence and attention. They found that, in general, representational paintings were preferred over abstract paintings. Correlating brain activity (via fMRI) with aesthetic preference, the researches demonstrated that activation in the right caudate nucleus decreased with decreasing preference, while the activation of fMRI signals in bilateral occipital gyri, left cingulate sulcus and bilateral fusiform gyri, all increased in response to increasing preference. These results imply that, because abstract art is less preferred by the observer, there is less reward, less emotional valence and reduced attention, all of which results in reduced brain activity.

It has been claimed that during the processing of art works, two different aspects take place—the processing of pictorial content and the processing of the artistic style (Cupchik et al., 1992; Augustin et al., 2008). In an event related potential (ERP) study, Augustin et al. (2011) found that processing of style starts later and develops more slowly than the processing of content (50 ms vs. 10 ms, respectively). They attribute this time difference in processing of the artwork to the fact that classification of content is extremely over-learned by humans as part of daily object classification and recognition whereas style analysis is a visual task that many have hardly ever experienced. They suggest (after Leder et al., 2004), that stylistic information might be processed as an abstract entity, which requires some high level processing, rather than a combination of low level embedding of features. This work also supports the notion that style specific information and art experience would facilitate and influence the perception of abstract art (more than of representational art). If this is indeed the case then abstract art, which exposes us mostly to the style of work and hardly to a significant content of it (as no particular objects are depicted), is being processed mostly via brain’s routes of style analysis; routes that are less familiar to, and less used by, most people. In other words, abstract art introduces us to unfamiliar (or less familiar) situation.

It should be noted that many of the brain imaging studies on art rely on “reverse inference”, that is to say that an activation of a particular brain area is used as an indication for the engagement of that brain’s area in a particular cognitive process. Whereas activity of a particular brain area during a specific cognitive process imply the involvement of that area in that cognitive function, the reverse proposition needs a wider support, via high selectivity of the response of that particular brain area, or increase in prior probability of the particular cognitive process (Poldrack, 2006).

Another feature that might be enhanced while looking at abstract art is how global is the pattern of observation when concrete recognizable objects are missing in the pictorial scene. Such lack of objects enables a more uniform global gaze. For example, Taylor et al. (2011) investigated eye tracking of viewers appreciating Jackson Pollock’s paintings, showing that the viewers’ eyes tend to scan rather uniformly the surface of the whole canvas. This finding is in clear contrast to, by now classical, eye tracking studies of representative art, whereby the eye teds to gaze mostly on salient features in the painting (e.g., eyes, nose, trees, signature, etc.) and to almost completely neglect the rest (majority) of the painting’s surface (see for example Locher et al., 2007; Hari and Kujala, 2009). The work of Taylor et al. (2011) supports the notion that, while analyzing abstract art, the visual/perception system is less engaged with focal and converging gaze but rather to a more homogeneous gaze. Again, a less familiar situation in our daily experience (see related work by Zangemeister et al., 1995). Another research found that in representational art, the eyes fixate longer on the figurative details than in abstract paintings, probably due to the lack figurative elements in the pictorial scene. This holds for both experts and laypersons (Pihko et al., 2011).

## Speculations regarding our attraction to abstract art

Pictorial art analysis can be regarded as composed of three main processes; (i) the brains’ effort to analyze the pictorial content and style; (ii) the flood of associations evoked by it; and (iii) the emotional response it generates (Bhattacharya and Petsche, 2002; also see Freedberg and Gallese, 2007). Of course, being man-made for no immediate practical use, art in general enables the viewer to exercise a certain detachment from “reality” which, so it seems, provides certain rewards to the art-lover.

But abstract art offers a particularly unique opportunity that is evoked by visual stimulus which is not object-related and, therefore, remote from our daily visual experience. This frees us, to a large extent, from (automatically) activating object-related systems in the brain whose task is to “seek” for familiar (memory-based) compositions. Such “survival” mechanisms (e.g., “binding” and “figure ground separation”) are not activated via abstract art, thus enabling us to form new “objects-free” associations that may arise from more rudimental visual features such as lines, colors and simple shapes. This conclusion is supported by both the lack of specific brain region(s) for the processing of abstract art exclusively (Kawabata and Zeki, 2004) as well as by the eye tracking experiments (Taylor et al., 2011), demonstrating that in abstract art, the eye (brain) is “free” to scan the whole surface of the painting rather than “fall” mostly into well recognized salient features, as is the case when processing representational art. Abstract art may therefore encourage our brain to respond in a less restrictive and stereotypical manner, exploring new associations, activating alternative paths for emotions, and forming new possibly creative links in our brain. It also enables us to access early visual processes (dealing with simple features like dots, lines and simple objects) that are otherwise harder to access when a whole “gestalt” image is analyzed, as is the case with representational art.

If indeed the above hypothesis were correct, then one would expect a larger variability of individual response between people, and at different times for same viewers, in brain response to abstract art as compared to representational art. Indeed, such variability was found by behavioral studies. Reflecting inner state rather than obeying to the dominance of visual objects, the response to abstract art is expected to be more dependent on one’s particular inner state at a very specific moment, more so than while observing representational art (which more automatically activates the “survival”-related brain system). At some instances, a particular abstract artwork might evoke strong association and emotional response than in other times, when the inner state of the viewer is less approachable, less amenable to processing abstract art. A related prediction is that abstract art would activate more of the default system in the brain, associated with inner-oriented processing. This prediction goes along with the findings of Cela-Conde et al. (2013), which demonstrate the involvement of the default mode network during the later phase of aesthetic appreciation. Relevant to the current paper is the claim expressed in the mentioned article, indicating the complex relations between the inner thoughts and the processing of external events (for more on the role and involvement of the default system in art appreciation see also Vessel et al., 2012; Mantini and Vanduffel, 2013).

In contrast, representative art would activate the extrinsic system more powerfully, as this system is associated with processing information arriving from the external environment (Golland et al., 2008).

To conclude—abstract art is a very recent (100 years old or so) invention of the human brain. Its success in attracting the brains of so many of us suggests that it has an important cognitive/emotional role. Supported by recent experimental studies, I claim that abstract art frees our brain from the dominance of reality, enabling the brain to flow within its inner states, create new emotional and cognitive associations and activate brain-states that are otherwise harder to access. This process is apparently rewarding as it enables the exploration of yet undiscovered inner territories of the viewer’s brain.

## Conflict of interest statement

The author declares that the research was conducted in the absence of any commercial or financial relationships that could be construed as a potential conflict of interest.
